# Isolation and Molecular Characterization of *Peste des Petits Ruminants Virus* from Outbreaks in Southern Ethiopia, 2020

**DOI:** 10.1155/2022/5329898

**Published:** 2022-05-29

**Authors:** Abde Aliy Mohammed, Tesfaye Rufael Chibssa, Waktole Terfa, Fasil Aklilu, Delesa Damena, Redeat Belayneh, Menbere Kidane

**Affiliations:** ^1^National Animal Health Diagnostic and Investigation Center (NAHDIC), P.O. Box 04, Sebeta, Ethiopia; ^2^Ambo University, School of Veterinary Sciences, P.O. Box 19, Ambo, Ethiopia

## Abstract

Peste des petits ruminants (PPR) is one of the most important transboundary diseases of small ruminants. In this study, nasal and oral swabs (*n* = 24) were collected from sheep (*n* = 7) and goats (*n* = 17) with clinical signs in southern Ethiopia in March 2020. PPR virus was isolated on Vero dog cells expressing the signaling lymphocyte activation molecule (VDS) and screened using RT-qPCR. Positive samples were confirmed by conventional RT-PCR followed by sequencing of a partial nucleoprotein (N) gene segment. Results revealed that 54% (*n* = 13/24) of the tested samples were PPRV-positive Phylogenetic analysis revealed that the viruses belonged to lineage IV and lineage II. The lineage IV viruses were similar, although not identical, to other lineage IV viruses previously reported in Ethiopia and other East African countries while the lineage II viruses have been reported for the first time in Ethiopia showed a high nucleotide identity (99.06%) with the vaccine (Nigeria 75/1) that is currently used in Ethiopia for the prevention of PPR. Further investigations are therefore recommended in order to fully understand the true nature of the lineage II PPRVs in Ethiopia.

## 1. Introduction

Peste des petits ruminants (PPR), also known as sheep and goat plague, is a highly contagious animal disease affecting domestic and wild small ruminants [1]. PPR is a highly contagious transboundary viral disease affecting mainly goats and sheep, as well as dromedaries. Long overlooked, it is now present in most countries of Africa, the Near and Middle East, and Asia, causing considerable losses in livestock. Despite the existence of a highly effective vaccine, PPR continues to spread geographically. The illegal movement of infected animal products represents a further potential threat for disease spread to PPR free countries [2].

In rural areas of Ethiopia where sheep and goats are important for livelihoods, the disease has a significant impact on local economies [3]. PPR is caused by the Peste des petits ruminants virus (PPRV), also known as small ruminant morbillivirus, is a member of the Morbillivirus genus, family Paramyxoviridae, order Mononegavirales [4]. The genome of PPRV encodes 8 proteins, including two non-structural proteins C and V, a nucleoprotein (N), a viral RNA-dependent polymerase (L), an RNA-polymerase phosphoprotein cofactor (P), a matrix protein (M), a fusion protein (F), and a hemagglutinin protein (H) [5]. PPRV is classified into four lineages (I–IV) based on the genetic comparison of a fragment of the nucleoprotein or the fusion protein [6]. The extensive detection of lineage IV virus in Africa suggests that it is replacing other lineages across different areas [7–9].

In Ethiopia, lineage IV is slowly replacing PPRV lineage III [10, 11]. Because of its economic burden in many developing countries, PPR is a priority animal disease that has been targeted for global eradication by 2030 by the Food and Agriculture Organization of the United Nations (FAO) and the World Organization for Animal Health (OIE) [12]. In Ethiopia, PPR affects small ruminant production and contributes to food insecurity, particularly in pastoral regions due to its potential for rapid spread and associated restrictions on the international trade of animals and animal products [8, 13–15].

Borena zone is a pastoral area in southern Ethiopia where PPR disease outbreaks have been frequently reported. However, the circulating PPRVs have not been well characterized [16]. The objective of this study was to isolate and characterize the currently circulating PPRV strains obtained from outbreak cases in the Borena zone.

## 2. Material and Methods

### 2.1. Study Area

**Figure 1:**
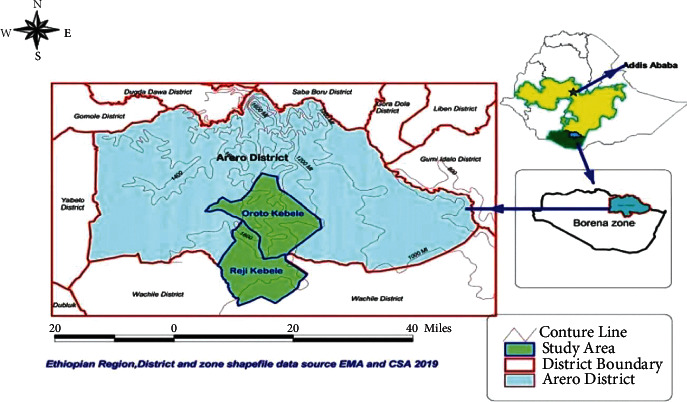
Map of Arero district showing the location of outbreaks of the study areas.

The study was conducted in March 2020 in the Arero district (Oroto and Reji) of Borena zone, in southern Ethiopia. The Arero district is a pastoral livestock production system with communal grazing (Figure 1). The climate is semiarid, which receives annual average rainfall ranging from 500 mm^3^ to 700 mm^3^. Delivery of the rainfall is bimodal: 56% of the annual rainfall occurs from March to May and 27% from mid-September to mid-November [17]. The annual mean daily temperature varies from 19°C to 24°C with moderate seasonal variation. The livestock populations are approximately 1.7 million cattle, 2 million sheep and goats, 700,000 camels, and 64,000 equines [18]. The Arero pastoralists manage their dominant animal species, in a traditional pastoral system that is driven long distances in search of good pasture and surface water, irrespective of national boundaries [17].

### 2.2. Study Methodology

The outbreak investigation was conducted in March 2020 in Borena zone. Sheep and goats with clinical signs of high fever, diarrhea, and nasal and ocular discharge were sampled. A total of 24 swabs (oral and nasal) were collected from two suspected PPR outbreaks (Table 1). The collected swab samples were transferred into sterile vials containing virus transport media (VTM) which contained phosphate-buffered saline (PBS), appropriately labeled, kept chilled using ice packs, transported to NAHDIC, and stored at −80°C for further laboratory analysis.

### 2.3. Laboratory Analysis

#### 2.3.1. Virus Isolation

The virus isolation was conducted in Vero Dog SLAM cells (VDS) as outlined in the OIE manual [19]. Briefly, the swab samples were homogenized and centrifuged at 3000 rpm for 20 minutes at 4°C and the supernatant was used for inoculation of VDS cells grown on 24-well plates [20]. The inoculated cells were incubated at 37°C, 5% CO_2_, and 96% humidity for one hour with slight intermittent shaking at 15-minute intervals to allow adsorption of the virus. The virus inoculum was decanted followed by washing with Dulbecco's modified Eagle's medium (serum-free DMEM) to which 500 *μ*l of maintenance medium (DMEM with 2% fetal bovine serum heat inactivated-Gibson) and amphotericin B were added. The inoculated cells were observed under an inverted microscope for any nonspecific reactions and incubated at 37°C and 5% CO_2_ for 7 days. The cells were then monitored daily under an inverted microscope for cytopathic effects due to viral replication [21].

### 2.4. Polymerase Chain Reaction (PCR) Tests

#### 2.4.1. RNA Extraction

Viral RNA was extracted from the swab samples using the QIAamp Viral RNA Mini Kit (Qiagen, Hilden, Germany) following the manufacturer's guidelines. The eluted viral RNAs were stored at −80°C for further testing.

#### 2.4.2. Real-Time PCR (RT-qPCR)

The RT-qPCR assay was performed on an Applied Biosystems 7500 thermal cycler for all extracted viral RNA using specific primers and a probe for the N gene as described in [22]. Briefly, the RT-qPCR was performed in a final reaction volume of 20 *μ*l containing: 10 *μ*l of Express Universal superscript (Invitrogen), 0.5 *μ*l of superscript enzyme, 0.5 *μ*l of passive reference Rox, 1.5 *μ*l of each primer PPRV forward primer (5′AGA GTT CAA TAT GTT RTT AGC CTC CAT 3′), PPRV reverse primer (5′TTC CCC ART CAC TCT YCT TTGT 3′) and 1.0 *μ*l of PPR probe (FAM-CAC CGG AYA CKG CAG CTG ACT CAG AA-QSY), 2 *μ*l of RNAse-free water, and 3 *μ*l of RNA template. The amplification was performed at 50°C for 15 min, 95°C for 20 seconds, followed by 45 cycles of denaturation and annealing at 95°C for 3 seconds and extension at 60°C for 30 seconds. The samples that had a *Ct* value < 35 were considered positive [22].

### 2.5. Conventional RT-PCR

Conventional RT-PCR was conducted following a standard method as described in the OIE manual [19] using primer pairs: NP3 (5′GTC TCG GAA ATC GCC TCA CAG ACT 3′) and NP4 (5′ CCT CCTCCT GGT CCT CCA GAA TCT 3′) [23]. The amplification was carried out in a final reaction volume of 25 *μ*l containing 7.5 *μ*l of RNase-free water, 5 *μ*l of 5XRT-PCR buffer (Qiagen), 1 *μ*l of deoxyribonucleotide triphosphate (dNTP), 5 *μ*l of *Q* solution, 1.5 *μ*l of each primer NP3 forward and primer NP4 reverse, 1 *μ*l of one-step enzyme mix and 2.5 *μ*l of RNA template at 50°C for 30 min, 95°C for 15 min, followed by 40 cycles of denaturation at 94°C for 30 s, annealing at 60°C for 30 s, extension at 72°C for 1 min, and final extension at 72°C for 5 min in an Applied Biosystems 2720 thermal cycler. The PCR products were analyzed by gel electrophoresis on a 1.5% (w/v) agarose gel [23].

### 2.6. Partial N Gene Sequencing and Sequence Analysis

The amplified positive PCR products were sequenced at Pennsylvania State University, USA. The sequence data were assembled using Vector NTI 11.5 software (Invitrogen, USA). Nucleotide sequences were aligned using the ClustalW algorithm implemented in BioEdit 7.1 [24]. The partial N gene sequences (351 bp) of eight sequences from this study and additional sequences from other countries in Africa and Asia were retrieved from GenBank and included for comparative analysis. For phylogenetic reconstructions, the sequences were aligned with the Muscle algorithm (codon option) implemented in BioEdit 7.1. The phylogenetic tree was constructed using the maximum-likelihood (ML) and 1000 bootstrap replications in MEGA6 [25].

### 2.7. Ethical Approval

This research study was approved by the Animal Research Scientific and Ethics Review Committee (ARSERC) of the National Animal Health Diagnostic and Investigation Center (NAHDIC), Sebeta, Ethiopia, for nonexperimental research (Certificate Ref. No: ARSERC/EC/014/22/12/2020). Guidelines for the care and use of animals were followed, and oral consent was acquired from animal owners prior to sampling their animals.

## 3. Results

### 3.1. Clinical Observation

High fever, nasal discharge, crusts around the nostrils, lachrymal discharge, ulcerative oral lesion, and diarrhea were observed in the affected sheep and goats. The results indicated morbidity of about 80% and mortality of 45% in this study.

A total of 24 swabs (nasal and ocular) samples were collected according to Table 1. Out of this, 6 (25%) samples were positive by virus isolation and 13 (54%) samples were positive by RT-qPCR with *Ct* values ranging from 17 to 30. Ten of the RT-qPCR-positive samples were confirmed by conventional RT-PCR.

### 3.2. Virus Isolation

**Figure 2:**
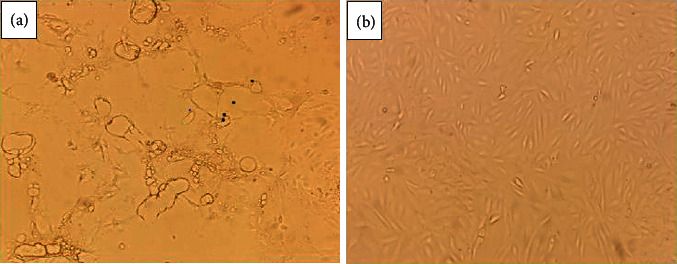
Virus isolation in VDS cell culture. (a) Postinfection with PPR virus showing a vacuolation, aggregation, and syncytia formation. (b) VDS cell culture as the negative control.

Six out of 24 samples attempted for virus isolation were recovered (Figure 2). The cytopathic effect (CPE) in VDS cells developed vacuolation, aggregation or clustering together, and syncytia formation of the cells (Figure 2(a)). Negative control (PBS-inoculated) cell culture did not show any CPE (Figure 2(b)).

### 3.3. Detection of PPRV by Real-Time and Conventional PCR

**Figure 3:**
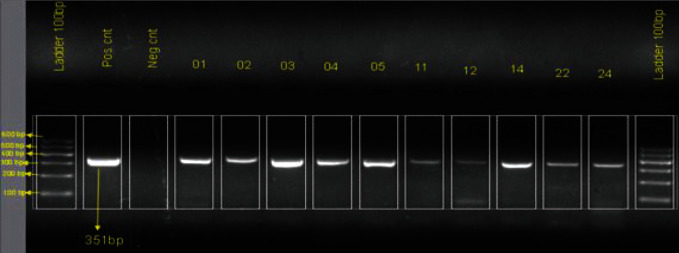
Conventional PCR-amplified products of 10 positive samples showing a band size of 351 bp of viral N gene (from left to right). The first lane is the DNA ladder of 100 bp, and the second and third lanes are the positive and negative control, respectively. Lane represented by nos. 01, 02, 03, 04, 11, 12, 14, 22, and 24 are study samples.

Of a total of 24 clinical samples examined, 54.1% were positive by real-time RT-PCR for viral nucleic acid, with *Ct* values ranging from 17 to 30. To confirm the amplification by RT-PCR, conventional PCR was performed to check the quality of band and size of the fragments and the expected band was observed at 351 bp fragment size and no band of negative control (Figure 3).

### 3.4. Sequencing and Phylogenetic Analysis

All 10 amplicons from the conventional RT-PCR-positive samples were sequenced. However, two samples obtained from Reji did not generate the sequence. The remaining eight N gene nucleotide sequences of PPRV PCR amplicons obtained in this study were submitted to GenBank under accession numbers OL690338 to OL690345.

**Figure 4:**
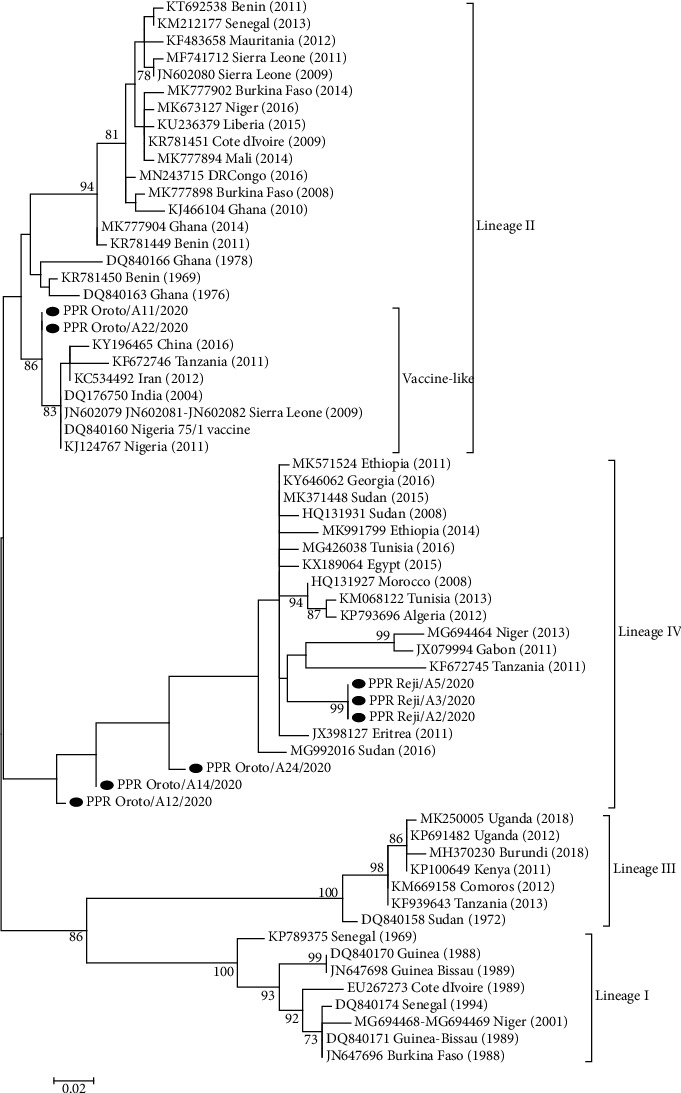
Phylogenic tree based on the partial sequence of N gene (351 bp) PPRV isolates. The tree was constructed using the maximum-likelihood (ML) method [25]. The PPRV isolate from this study is indicated by filled black circles.

In order to represent a comprehensive picture of PPRV circulating in the region, phylogenic tree construction was performed based on N gene-obtained sequences in this study and collected a list of various sequences reported to GenBank (Figure 4).

The phylogenetic tree based on the N gene of PPRV showed that all eight sequenced isolates in this study belong to PPRV. The consciences tree has four fixed genetic clusters consisting of I, II, III, and IV lineages (Figure 4).

Phylogenetic reconstruction, of six sequences from viruses PPR-Reji/A2/2020, PPR-Reji/A3/2020, PPR-Oroto/A12/2020, PPR-Reji/A5/2020, PPR-Oroto/A14/2020, and PPR-Oroto/A24/2020, showed that they clustered in lineage IV. The sequences of the viruses from Reji were identical to each other and were related to other PPRV sequences obtained in Eastern Africa (e.g., Eritrea, Sudan, and Tanzania). PPR-Oroto/A12/2020, PPR-Oroto/A14/2020, and PPR-Oroto/A24/2020 are very interesting because they are only 93–94% identical to the closest PPRV isolated from wildlife in Sudan (MG992016 Sudan-2016). Two PPRV sequences, Oroto/A11/2020, and PPR_Oroto/A22/2020 are grouped within lineage II, which is the first time that lineage II has been reported in Ethiopia (Figure 4). These two sequences reviled a high nucleotide identity (99.06%) to the Nigeria 75/1 vaccine strain.

## 4. Discussion and Conclusion

There have been numerous outbreaks of PPR reported in Ethiopia, most often confirmed by the observation of characteristic clinical symptoms and serological tests. The results indicated morbidity of about 80% and mortality of 45% in this study. Since 1994, a number of PPR outbreaks have been reported in different regions of Ethiopia with variable morbidity and mortality [10, 26].

To understand the molecular epidemiology of these PPR outbreaks in Ethiopia, it is important to perform isolation and genetic characterization of PPR viruses in order to develop effective prevention and control strategies in the region. In this study, the presence of the PPR virus was demonstrated, by viral isolation and molecular characterization. The present study uses Vero Dog SLAM (VDS) cells that were suitable for the isolation of PPRV from the field samples, as SLAM protein is used for a cellular receptor as outlined in the OIE manual [19].

Phylogenic analysis revealed that six of the viruses identified (e.g., PPR-Oroto/A2/2020, PPR-Oroto/A3/2020, PPR-Oroto/A12/2020, PPR-Oroto/A5/2020, PPR-Oroto/A14/2020, and PPR-Oroto/A24/2020) clustered with other members of PPRV lineage IV sequences. This result is consistent with earlier findings indicating that lineage IV is circulating in Ethiopia [10, 11, 27]. They are also closely related to isolates from neighboring countries such as Sudan (MG992016) and isolates from Eritrea (JX398127). This study is interesting, as it has proven a potential for wildlife reservoir of PPR in the East Africa region. The study also similarly indicated that lineage IV is also circulating widely in East Africa such as Sudan, Eretria, and Uganda as well as Egypt [6, 9, 28].

Interestingly, from the phylogenetic tree, two of the viruses (Oroto/A11/2020 and Oroto/A22/2020) belong to lineage II, which is the first report of lineage II to our knowledge in Ethiopia. However, these viruses do not cluster with lineage II viruses that are currently circulating, primarily, in West Africa. Instead, they are highly similar (99.06% nucleotide identity) to the lineage II virus (Nigeria 75/1) currently being used as an inactivated vaccine in Ethiopia. It must be questioned whether these viruses are truly circulating in the country or whether they are the result of detecting viral replication of Nigeria 75/1 vaccinated animals. Indeed, similar reports of the detection of vaccine-like lineage II viruses in the field have been published in China, Iran, India, Sierra Leone, Tanzania, and Nigeria [29–31] and there is ongoing debate as to whether they are not just due to laboratory contamination. Further studies which should include vaccination studies are required to clarify these findings.

This study's finding of lineage II PPRV may not be unexpected as PPRV was known to be circulating in neighboring countries. It is, therefore, important to characterize PPRV strains in the East Africa region with emphasis on countries such as Somalia, Djibouti, Kenya, Uganda, Tanzania, S. Sudan, and Sudan to understand and trace the movement of virus and animals between countries so that regional eradication of this devastating disease may be achieved [29].

In spite of being endemic in Ethiopia, outbreaks of PPRV are regularly occurring, and limited information on the genetic nature of PPRV is known. Our current data demonstrate that PPRV lineage IV is still circulating and causes economic losses in small ruminant's production in Ethiopia. Identification of PPRV lineage II for the first time in Ethiopia described in this study can increase the available information on the circulating PPRV strains in Ethiopia. To show the complete picture of the circulating PPRV in the country, continuous monitoring and surveillance of the situation of the PPRV in Ethiopia including East Africa Countries are needed. Therefore, a proper understanding of this virus circulation will help PPRV endemic countries such as Ethiopia to have global PPRV eradication campaigns.

## Figures and Tables

**Table 1:** Description of sample used and test results by different test methods in this study.

Outbreak area	Sample number	Species		No. of positives for different test methods			
		Goats	Sheep	Virus isolated	Real-time PCR	Conventional PCR	Partial N gene sequence
Reji	10	6	4	3	5	5	5
Oroto	14	11	3	3	8	5	5
Total	24	17	7	6	13	10	10

## Data Availability

No supporting data are available.
